# Green Natural Rubber Composites Reinforced with Black/White Rice Husk Ashes: Effects of Reinforcing Agent on Film’s Mechanical and Dielectric Properties

**DOI:** 10.3390/polym13060882

**Published:** 2021-03-13

**Authors:** Praewpakun Sintharm, Muenduen Phisalaphong

**Affiliations:** Bio-Circular-Green-Economy Technology & Engineering Center, BCGeTEC, Department of Chemical Engineering, Faculty of Engineering, Chulalongkorn University, Bangkok 10330, Thailand; praewpakun.s@gmail.com

**Keywords:** natural rubber, rice husk ash, alginate, mechanical properties, dielectric properties

## Abstract

Green natural rubber (NR) composites reinforced with black rice husk ash (BRHA)/white rice husk ash (WRHA), using alginate as a thickening and dispersing agent and crosslinking by CaCl_2_, was developed to improve mechanical, chemical and dielectric properties of NR-based films by using a latex aqueous microdispersion process. A maximum of 100 per hundred rubbers (phr) of rice husk ashes (RHAs) could be integrated in NR matrix without phase separation. Mechanical properties of the composite films were considerably enhanced, compared to the neat NR film. The composite films reinforced with WRHA demonstrated relatively better mechanical properties than those reinforced with BRHA, whereas the composites filled with BRHA demonstrated higher elongation at break. The crosslinking by CaCl_2_ improved the film tensile strength but lowered the film elasticity. The reinforcement strongly improved chemical resistance of the composite films in toluene. The films are biodegradable in soil, with weight loss of 7.6–18.3% of the initial dry weight after 3 months. Dielectric constant and dielectric loss factors of the composite films were enhanced with RHAs loading. According to the obtained properties, the composites offer potential for further development as stretchable conductive substrate or semiconducting polymer films for electronic applications.

## 1. Introduction

In recent years, biopolymers have attracted considerable interest for their potential to reduce consumption of fossil resources and nonbiodegradable polymers in order to reduce plastic pollution. Biopolymers are usually biodegradable, meaning that they can be decomposed and degraded into CO_2_ and water by microorganisms in the environment. Recent research has focused on improving properties of biopolymer composites, including starch-based materials, cellulose-derived polymers, bacterial polyesters and natural rubber composites. The blending process, which involves adding biofillers (such as agricultural waste, cellulose and ash), can be used to modify the properties of biopolymer composites [1]. Biodegradable polymers can be used in many electronics applications; for instance, they can be used as insulators, (semi)conductors, dielectric material or electronic packaging [2].

Thailand is the world’s largest producer and exporter of natural rubber (NR). In 2017, over 4.5 million tons of NR were produced in the country (about 37% of global production) [3]. NR is exported in several basic forms, including ribbed smoked sheets, concentrated latex and compound rubber. However, the NR prices have been unstable for several years, because of oversupply from major rubber producing countries, a weak global economy and cheaper synthetic rubbers for replacement natural rubber [3]. In order to increase the value of NR, technologies for improving NR properties or transforming NR latex into higher value products have to be developed. NR is one of the important elastomers and is widely utilized to prepare many rubber products such as vehicle tires, belts, condoms, gloves, health equipment and accessories, coatings and floor covering [4]. NR is nonsaturated rubber, because its structural unit contains a double bond. Thus, it has very low aging resistance, because ozone and oxygen can react with the double bond very easily. Consequently, NR often has its chemical structure modified by a vulcanization process and reinforced by adding fillers to improve its properties [5]. Carbon black and silica are synthetic fillers that are commonly used as reinforcing fillers in the rubber industry. Both carbon black and silica are added into an NR matrix in order to improve the mechanical and thermal properties of NR composites. However, the major problem of this type of reinforcement is the poor dispersion and agglomeration of fillers in the NR matrix, which limits the loading content of fillers.

Natural fillers are becoming more popular as alternative materials for NR reinforcement, because they are biodegradable, environmentally friendly, cheap to purchase and help to reduce domestic waste [6]. In agricultural countries, rice husk is one of the major agricultural residues generated from the rice milling process and is usually used as a biomass fuel in steam boilers to generate steam in the power plant and rice milling plant. Burning rice husk as fuel results in the production of rice husk ash (RHA) as a waste material [7]. There are two types of RHA, white rice husk ash (WRHA) and black rice husk ash (BRHA). WRHA is obtained from the combustion of rice husk in the atmosphere, whereas BRHA is from the pyrolysis of rice husk in a poor oxygen atmosphere [8]. Usually, rice husk ash waste is released into the environment without any commercial return. Because rice husk ash is usually composed of silica (SiO_2_) (over 80–90%) and carbon black, it is attractive for use as reinforcing filler for NR composites. Previous studies have reported on NR composites produced from NR and mixed rice husk ash in solid state using a two-roll mill (the conventional process) [9,10,11]; however, the limitations of this method include poor NR matrix–filler interaction and low dispersion of fillers in NR composite [11,12,13,14,15]. A new latex compounding method was developed to solve the problem of incompatibility between NR and fillers and improve the dispersion of fillers in the NR matrix [16,17]. Moreover, the latex compounding method consumes less energy during mixing when compared to the conventional process [18].

The aim of this study is to improve the mechanical, chemical and dielectric properties of uncured NR composite films prepared via a latex aqueous microdispersion process through the addition of different types of rice husk ashes (RHAs). In order to obtain good dispersion of polar fillers in a nonpolar NR matrix, sodium alginate, one of the most commonly used natural polysaccharides, was employed as a thickening and dispersing agent in the mixture. Through the use of this technique, high concentrations of fillers can be added into the NR matrix without phase separation. The effects of reinforcing NR composites with RHAs and crosslinking of the dispersing agent (alginate) by CaCl_2_ on the chemical, mechanical, biodegradation and dielectric properties were investigated for further development of the composite films.

## 2. Materials and Methods

### 2.1. Materials

Rice husk and rice husk ash were obtained from a rice milling plant in Phra nakhon si Ayutthaya province, Thailand. NR latex with a 60% dry rubber content was purchased from the Rubber Research Institute of Thailand, Bangkok, Thailand. Hydrochloric acid, sodium alginate and calcium chloride were purchased from Sigma-Aldrich (Thailand) Co Ltd., Bangkok, Thailand.

### 2.2. Methods

#### 2.2.1. Preparation of Black Rice Husk Ash (BRHA) and White Rice Husk Ash (WRHA).

BRHA used in this study was kindly provided by Nakhon Luang Rice Mill (Phra Nakhon Si Ayutthaya, Thailand), in which the combustion process was under oxygen-depleted atmosphere due to the limited air supply. BRHA from a rice milling plant was dried in an electric oven at 105 °C for 12 h to remove water and stored in a glass bottle container at room temperature (~30 °C). WRHA was prepared in our laboratory (Chulalongkorn University, Bangkok, Thailand). Initially, rice husks were washed with distilled water to remove dirt and impurities and then were dried in an oven at 105 °C for 24 h. The dried rice husks were submitted to the heat treatment in ceramic crucibles, which was carried out in an atmosphere of air in an electric furnace at the temperature of 500 °C for 2 h [19]. The particle sizes of both types of rice husk ashes were reduced using ball milling (PM 100, Haan, Germany) at revolution speed 400 rpm for 15 min and sieved (Test sieve ASTM, 203 × 50 mm, 106 µm, 140 mesh) in order to obtain th2e powder with a size of less than 106 µm.

#### 2.2.2. Preparation of NR Composite Films.

The NR composite films were reinforced by using BRHA and WRHA as filler. The filler loading used in this work was varied at 0, 20, 60 and 100 per hundred rubbers (phr). The microdispersion process [16] was applied for the preparation of composite films. Initially, the filler was added into the aqueous solution of 1% *w*/*v* alginate (which was found to be a suitable solution for good dispersion of BRHA and WRHA). Then, the slurry was thoroughly mixed under mechanical stirring at room temperature for 30 min. Then, 5 g of 60 phr NR latex was slowly added into 30 mL of the slurry under continuous mixing by high-frequency mechanical stirring until the mixture was homogenous. After that, the mixture was poured into a plastic tray and dried overnight (~12 h) in an oven at 40 °C to obtain NR composite films. The composite films of NR combined with BRHA and WRHA are referred to as NR–B and NR–W, respectively.

#### 2.2.3. Crosslinking with CaCl_2_.

The aqueous solution of CaCl_2_ at 1% *w*/*v* was used as crosslinking agent. The solution was prepared by dispersing CaCl_2_ in distilled water and stirring at room temperature for 30 min. The NR composite films were soaked in 1% *w*/*v* CaCl_2_ solution for crosslinking of alginate for ~1 h, and then the films were rinsed with distilled water to remove excess chloride. After that, the composite films were dried in an oven overnight at 40 °C [20]. After crosslinking with Ca^2+^, the crosslinked composite films of NR combined with BRHA and WRHA are referred to as NR–BC and NR–WC, respectively.

### 2.3. Characterization

Morphologies of rice husk ashes and composite films were observed by scanning electron microscope and energy dispersive X-ray spectrometer (SEM–EDS) (IT-500HR) using a JEOL, JSM-IT-500HR and JEOL, JED-2300 (JEOL, Tokyo, Japan). The specimens were frozen in liquid nitrogen and vacuum dried. After that, the specimens were sputtered with gold. The SEM–EDS performed at an accelerating voltage of 10 kV.

The overall components and particle size distribution of each dried sample were determined by X-ray fluorescence spectrometer analysis (Bruker model S8 Tiger, Karlsruhe, Germany) and laser particle size distribution analyzer (Mastersizer 3000, Malvern Panalytical, Malvern, UK), respectively. The surface area, pore volume and average pore diameter of samples were determined by nitrogen (N_2_) physisorption-desorption using a surface area and pore size analyzer (Autosorb-iQ-MP, Quantachrome, Boynton Beach, FL, USA).

The structural information and crystallinity of fillers and NR composite films were characterized using X-ray diffractometer (XRD, Bruker AXS Model D8 Discover, Karlsruhe, Germany) with Cu-Kα radiation in the 2θ range of 5–40°. The operation conditions were at the accelerating voltage of 40 kV and electric current of 30 mA.

The functional groups and possible interaction between fillers were determined by Fourier transform infrared (FTIR) spectroscopy (PerkinElmer, Waltham, MA, USA) in the ranges of 4000–650 cm^−1^ with a resolution of 4 cm^−1^.

For mechanical properties tests of dry film of NR, NR composites (Young’s modulus, tensile strength and elongation at break) were performed using Universal Testing Machine (Instron, Norwood, MA, USA). The test conditions were according to ASTM D882. At least five specimens for each different blend composition were tested.

The measurement of water absorption capacity (WAC) was performed by using the specimen films of 2 × 2 cm^2^, with a thickness of 0.5–0.6 mm. The specimens were immersed in distilled water at room temperature for 0–18 days. The specimens were removed from water every 2 days, and excess water at the surface of the samples was blotted by Kimwipes^®^ paper. The weight of wet sample was measured. All testing was carried out in triplicate. Water absorption capacity was calculated by using the formula:

$$\mathrm{Water}\;\mathrm{absorption}\;(\%)\;=\;\frac{W_{h\;}-\;W_{d}}{W_{d}}\;\times 100$$

where, $W_{h}$ and $W_{d}$ are the weights of the hydrated and dried specimens, respectively.

For the test of toluene uptake, dried sample films (2 × 2 cm^2^), with a thickness of 0.5–0.6 mm, were immersed in toluene at room temperature. The weight change was monitored at 1 h intervals for 8 h. All testing was carried out in triplicate. Toluene uptake was calculated by using the formula:

$$\mathrm{Toluene}\;\mathrm{uptake}\;(\%)\;=\;\frac{W_{t\;}-\;W_{d}}{W_{d}}\;\times 100$$

where, $W_{d}$ and $W_{t}$ are the weights of the specimens before and after immersion in toluene at a time (*t*), respectively.

For the preliminary test of biodegradation in soil, each test specimen, having 5 × 5 cm^2^ and a thickness of 0.5–0.6 mm, was used for the measurement of biodegradation in soil. Potting soil purchased from a garden center (Bangkok, Thailand) was used for the experiment. The main composition of potting soil was loam soil, compost manure and coconut coir. The soil temperature was around 28–30 °C. The moisture content of the soil was around 50–60%. The specimens were weighed and buried in soil at a depth of 10 cm for 3 months under ambient conditions, where the temperature range was 24 to 35 °C. After 1, 2 and 3 months, the samples were removed from soil, washed with deionized water (DI)water, dried at 40 °C for 12 h and recorded for their weights. The degradation was evaluated by measuring the weight loss by using the formula:

$$\mathrm{Biodegradation}\;(\%)\;=\frac{W_{0}-W_{i}}{W_{0}}\times 100$$

where, $W_{0}$ and $W_{i}$ are the weights of the specimens before and after being buried in soil, respectively.

The dielectric constant and dielectric loss factors were measured directly by using impedance analyzer with a precision impedance analyzer (4294A, Agilent, Santa Clara, CA, USA) at room temperature. The measurements were done at varying frequencies ranging from 103–106 Hz. The samples were coated by silver paint as electrode on both sides before measurement.

## 3. Results and Discussion

### 3.1. Characterization of BRHA and WRHA Particles

Rice husk ash is a major by-product of the combustion of rice husk to generate heat for boilers in rice milling plants, in which the burning temperature is usually above 800 °C. BRHA results from the pyrolysis of rice husk in poor oxygen atmosphere, whereas WRHA results from the combustion of rice husk in atmospheric air. The results of XRF analysis for chemical compositions of BRHA and WRHA are shown in Table 1. The main components of BRHA and WRHA were found to be silica as silicon dioxide (SiO_2_), at 87.0% and 95.3%, respectively. The minor components were alumina oxide (Al_2_O_3_) and potassium oxide (K_2_O) and small amounts of CaO, P_2_O_5_, MgO, Fe_2_O_3_, SO_3_, MnO, ZnO and Rb_2_O. In addition, the unburned carbon or loss of ignition value of BRHA and WRHA were found to be about 7.8% and 0.1%, respectively. The color of the rice husk depends on amount of unburned carbon in the ash [21]; the reported unburned carbon contents of gray-RHA and WRHA were 2.4% and 1.4%, respectively. The product of WRHA from combustion treatment contains higher silica content than BRHA, but the unburned carbon content of WRHA was lower than that of BRHA.

**Figure 1:**
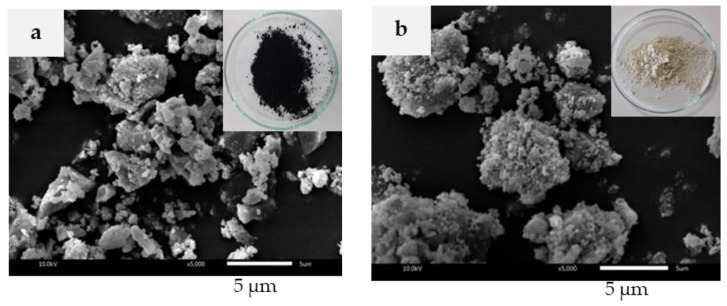
Scanning electron microscope (SEM) images of BRHA (**a**) and WRHA (**b**) particles.

The SEM images of BRHA and WRHA particles are shown in Figure 1a,b, respectively. BRHA formed black platelets of partially crystalline oxides of silicon and others, whereas WRHA was in roughly spherical form of aggregated white powders. Both ash particles were produced in a wide range of sizes, from 1 to 10 μm. However, the average size of BRHA was relatively smaller, compared to WRHA.

**Figure 2:**
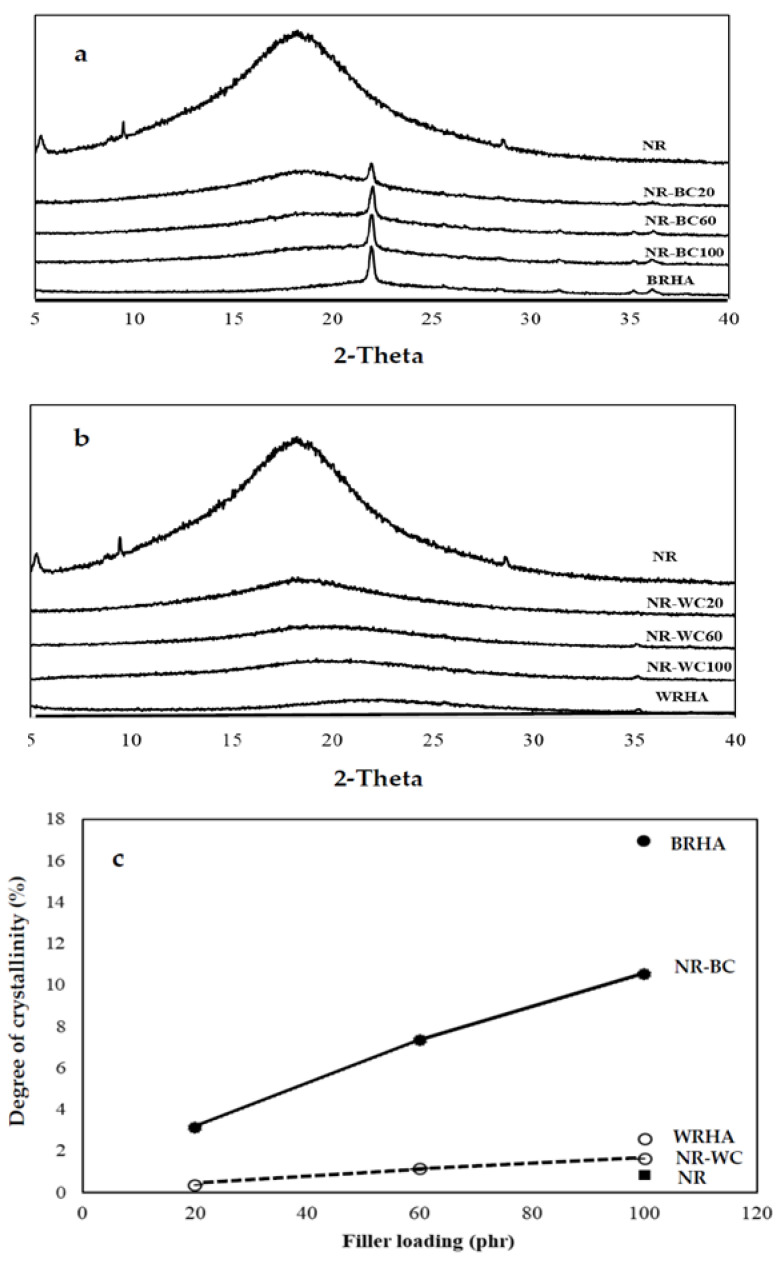
X-ray diffraction of the composite films of crosslinked composite films of natural rubber (NR) combined with BRHA (NR–BC) (**a**) and with WRHA (NR–WC) (**b**) and degree of crystallinity (**c**) with loaded content of BRHA and WRHA at 0, 20, 60 and 100 per hundred rubbers (phr).

The XRD analysis was conducted on BRHA and WRHA particles, as shown in Figure 2a,b. According to the XRD pattern, BRHA was in the form of partially crystalline oxides of cristobalite, corundum and quartz alpha. The observation of cristobalite and quartz alpha in BRHA indicates that BRHA should be treated at temperatures greater than 900 °C during the combustion process [22]. On the other hand, the XRD pattern of WRHA exhibited a very small peak of quartz alpha and broadening of the cristobalite peak (at 2θ = 22.5°), which indicates the nature of amorphous silica [23]. The degrees of crystallinity of BRHA and WRHA were 16.9% and 2.6%, respectively. The SEM micrographs and XRD results confirm that BRHA is a partially crystalline silica oxide, whereas WRHA is an amorphous silica oxide. These results agree with those reported by Xu et al. and Osman et al. [24,25], where WRHA with silica in a mainly amorphous form was produced at a controlled temperature below 800 °C, and BRHA with silica in partial crystalline phases was generated at temperatures above 800 °C.

**Figure 3:**
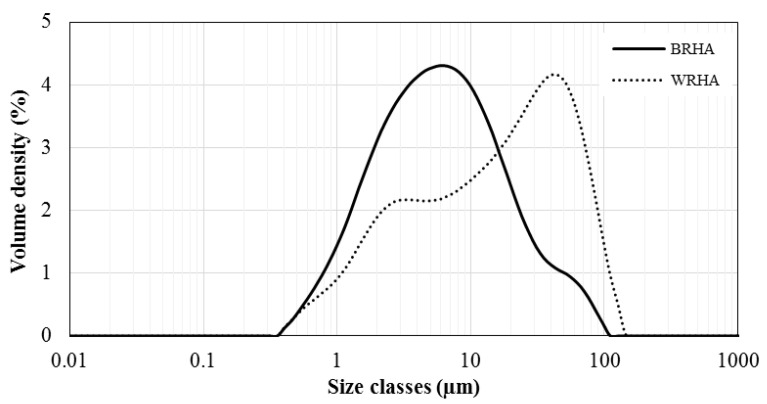
Particle size distributions of BRHA and WRHA particles.

The particle size distributions of the BRHA and WRHA are shown in Figure 3. The BRHA particles ranged in size from 0.4 to ~100 μm, with an average size ≈ 6 μm. The d10, d50 and d90 values based on the volume distribution were 1.5, 6.2 and 28.5 μm, respectively. The WRHA particles ranged in size from 0.4 to 144 μm, with an average size ≈ 40 μm. The d10, d50 and d90 of WRHA were higher than those of BRHA (1.9, 17.8 and 70.4 μm, respectively). The results from the SEM observation (Figure 1) and the particle size distribution (Figure 3) indicated that an average particle size of WRHA was larger than that of BRHA. This result can be attributed to hydroxyl groups of silanol (Si–OH) on the surface of rice husk ash particles. The hydroxyl group had very strong intermolecular hydrogen bonds with another hydroxyl of adjacent silica particle. These hydrogen bonds could easily cause the formation of agglomeration of particles. WRHA particles have larger particle sizes, as compared to BRHA particles, due to the higher degree of agglomeration of the WRHA powders, because of their higher silica content.

**Figure 4:**
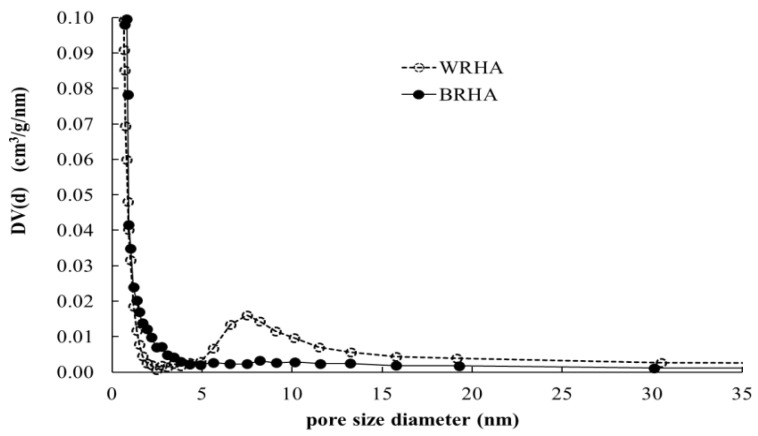
Pore size distributions of BRHA (●) and WRHA (○).

Pore size distributions of BRHA and WRHA are shown in Figure 4. Because WRHA formed agglomerated particles, WRHA presents bimodal pore structure of two groups of pores that are considerably different in size. The small pores inside of fine particle are less than 2.5 nm, and the pores between particles are 4–30 nm. On the other hand, BRHA shows a monodispersed mesoporous structure. Table 2 shows surface area, pore volume and pore size of BRHA and WRHA particles. Owing to the smaller particle size of BRHA, the specific surface area of BRHA (51.57 m^2^/g) was greater than that of WRHA (40.06 m^2^/g), as the decrease of particle size resulted in an increase in surface area. The average pore volume and average pore diameter of BRHA were lower than those of WRHA. The pore volumes of BRHA and WRHA were 0.24 and 0.48 cm^3^/g, respectively, with average pore diameter of 9.22 and 23.92 nm, respectively. These results can be attributed to larger pore size in WRHA agglomerated particles.

### 3.2. Morphology of NR and NR Composite Films

**Figure 5:**
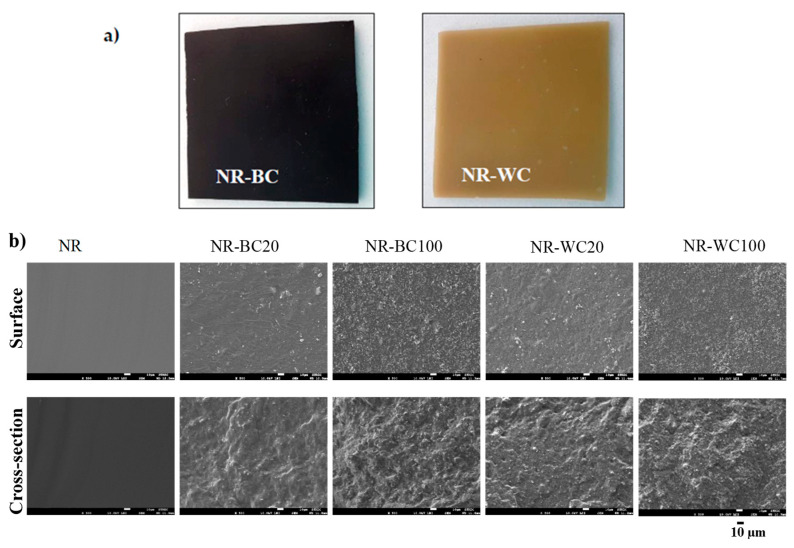
The outlook (**a**) and SEM micrographs (**b**) of surface morphologies (on the top) and cross-section (on the bottom) of NR, NR–BC20, NR–BC100, NR–WC20 and NR–WC100.

The morphologies of the NR, NR–BC and NR–WC specimens are illustrated in Figure 5. From the outlook (Figure 5a), both NR–BC and NR–WC composite films had a rather smooth surface. NR–BC films were black in color, whereas NR–WC films were light brown. The surface and cross-section areas of the neat NR film were smooth, whereas NR–BC and NR–WC had relatively rough surfaces. The degree of surface roughness increased with increases in BRHA and WRHA loading content. In addition, the cross-section area images show that both BRHA and WRHA demonstrated homogenous dispersion in NR matrix without phase separation. The results reveal that alginate is a good dispersing agent for the dispersion of rice husk ashes in NR matrix up to 100 phr.

### 3.3. X-ray Diffraction (XRD) of NR and NR Composite Films

As shown in Figure 2, the XRD pattern of pure NR film demonstrates a broad peak with very low degree of crystallinity (0.8%), which indicates an amorphous structure for the polymer phase. NR–BC and NR–WC composite films revealed diffraction peaks corresponding to BRHA and WRHA structures, respectively. In addition, the diffraction peaks of both ash composites increased linearly with increasing filler loading content. The degree of crystallinity of composite films also linearly increased along with the filler loading content (Figure 2c).

### 3.4. Fourier Transform Infrared (FTIR) Spectroscopy

**Figure 6:**
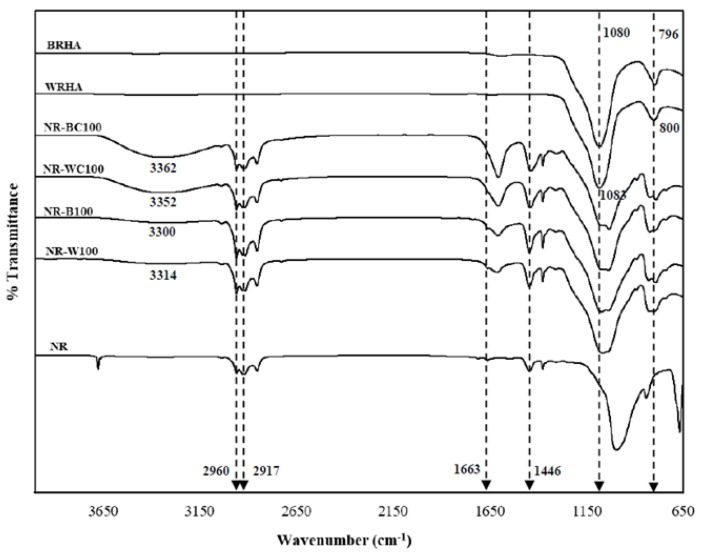
Fourier transform infrared (FTIR) of the WRHA; BRHA; NR; composite films of NR combined with BRHA (NR–B), NR–B100; composite films of NR combined with WRHA (NR–W), NR–W100; NR–BC100 and NR–WC100.

Figure 6 demonstrates the FTIR spectrum of NR, BRHA, WRHA, NR–B, NR–BC, NR–W and NR–WC composites. Pure NR consists mainly of cis-polyisoprene. The functional groups for identifying cis-polyisoprene were the asymmetric stretching vibration of methyl groups (–CH3) at 2960 cm^−1^ and C–H deformation at 1446 cm^−1^ [26]. The peaks observed at 2917 and 1663 cm^−1^ are assigned to symmetric stretching vibration of methylene (–CH2) and C=C stretching [17]. The BRHA and WRHA have similar FTIR spectrum. The strong sharp peaks at 1080 and 1083 cm^−1^ are attributed to Si–O–Si asymmetric stretching, along with peaks at 796 and 800 cm^−1^, which are due to symmetric Si–O–Si stretching and Si–O quartz [27,28]. The main broad peak between 1000 and 1200 cm^−1^ of WRHA indicates characteristics of amorphous silica [21]. The FTIR spectrum clearly indicates that functional groups found on NR–B, NR–W, NR–BC and NR–WC are the same as those found on NR, BRHA and WRHA. The spectra of the composites contain peaks at 2961, 2915, 1661 and 1067 cm^−1^, which are assigned to (–CH_3_), (–CH_2_), C=C stretching and Si–O–Si asymmetric stretching, respectively. The composite films reveal a broad absorption band between 3400 and 3100 cm^−1^, referring to the stretching of the O–H group. The hydroxyl regions of NR–BC and NR–WC are more dominant than those of the composite films without CaCl_2_ crosslinking, which might indicate that the CaCl_2_ crosslinking had an effect on the O–H interaction of sodium alginate (absorption band between 3500 and 3100 cm^−1^) [29]. Daemi and Barikani (2012) had previously reported that the absorption regions of O–H group in calcium alginate were narrower than the normal peak of sodium alginate, because the calcium ion decreased in hydrogen bonding between hydroxyl functional groups, resulting in narrower bands of calcium alginate [30]. The position peaks of functional groups of composite films are slightly shifted, as compared to the original spectra of NR, BRHA and WRHA, which indicates the formation of interactions of BRHA and WRHA in the NR matrix without a chemical reaction [16,17,20].

### 3.5. Mechanical Properties

**Figure 7:**
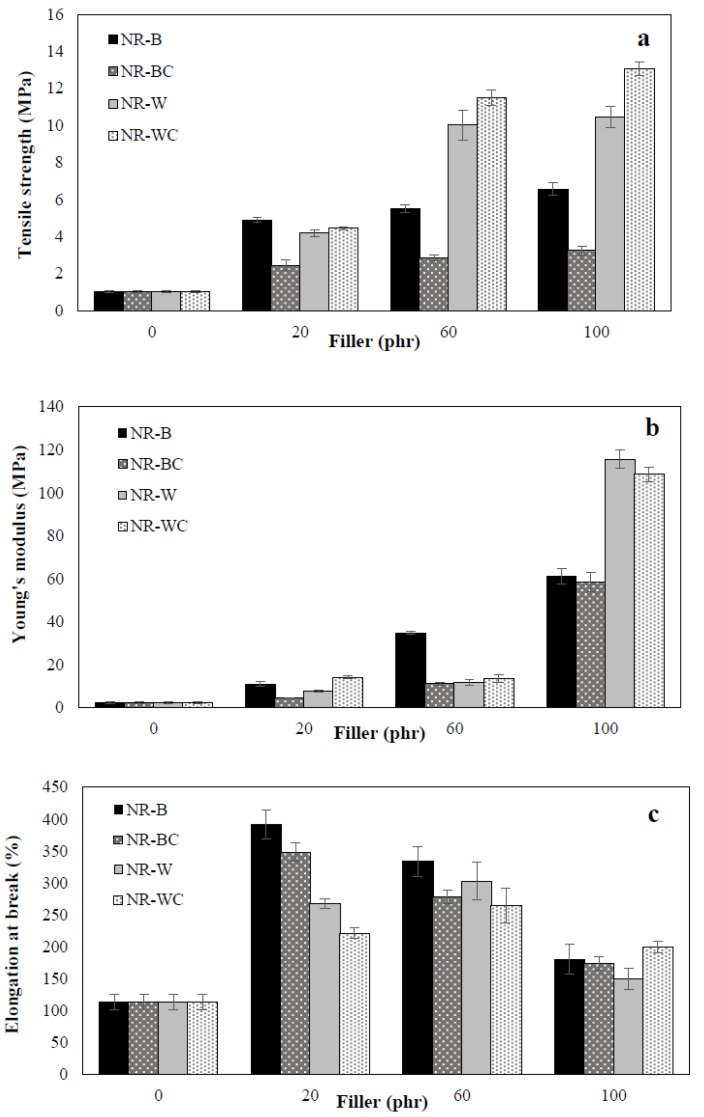
Mechanical properties, tensile strength (**a**), Young’s modulus (**b**) and elongation at break (**c**) of NR, NR–B, NR–BC, NR–W and NR–WC composite with filler loading content at 0, 20, 60 and 100 phr.

The mechanical properties of BRHA and WRHA composites were examined in terms of tensile strength, Young’s modulus and elongation at break, as shown in Figure 7. Uncured NR film was high elastic elongation, but it demonstrated low tensile strength. The tensile strength, Young’s modulus and elongation at break of the uncured NR film were 1 MPa, 2 MPa and 113.3%, respectively. Figure 7a clearly shows that the addition of BRHA and WRHA to the NR matrix resulted in improvement among all observed mechanical properties of the composite films. The tensile strength was relatively enhanced through the increase of BRHA and WRHA loading content in all cases. The maximum tensile strength values of the NR–B, NR–W, NR–BC and NR–WC were obtained by the filler loading at 100 phr, at 6.5, 10.4, 3.2 and 13.1 MPa, respectively. The results indicate that WRHA-filled NR films exhibit significantly higher tensile strength than those reinforced by BRHA, especially at filler loading content of 60 and 100 phr. The treatment of CaCl_2_ crosslinking tended to improve tensile strength of the composite films reinforced with WRHA but reduced the strength of the films reinforced with BRHA.

The reinforcement effects on Young’s modulus of composites filled with BRHA and WRHA were quite similar to those on tensile strength. The values of Young’s modulus were improved significantly, as compared to that of pure NR film, and the values increased with increasing filler content, as shown in Figure 7b. The maximum values of Young’s modulus of NR–B, NR–W, NR–BC and NR–WC films were obtained by BRHA and WRHA loadings at 100 phr, which were 61.1, 115.6, 58.4 and 108.6 MPa, respectively. As compared to BRHA, the reinforcement by WRHA was more effective in improving strength and modulus, which is likely due to the higher content of SiO_2_ in WRHA.

As compared to the result of the NR film shown in Figure 7c, it is demonstrated that reinforcement with BRHA and WRHA can also improve elongation at break of the composite films. The maximum values of elongation at break of NR–B at 392.0% and NR–W at 302.7% were obtained at filler loading of 20 phr and 60 phr, which were 3.5-fold and 2.7-fold increases of that of the NR film, respectively. However, the values relatively decreased with further increases in filler loading beyond the optimal point, because the elasticity of the rubber chains was reduced when filler particles exceeded a certain amount in the rubber matrix [31,32].

In the case of a small amount of filler in NR composite (20 phr), the tensile strength, Young’s modulus and elongations at break of composites filled with BRHA were better than those filled with WRHA. These results might be attributable to the smaller particle size of BRHA, since the smaller particle with higher specific surface area should facilitate a better filler—NR interaction [9]. However, when increasing filler loading in the NR composites to 60 and 100 phr, the tensile strength and Young’s modulus of NR–WRHA were greater than those of NR–BRHA. Thus, factors, such as surface activity, chemical composition of fillers, concentration, shape and interactions between fillers and NR matrix play important roles in the reinforcement of NR composites. As compared to BRHA, WRHA contains a higher concentration of silica but lower concentration of carbon. Higher concentration of silica and metal oxides in fillers could promote reinforcement in NR composites. Additionally, in this study, the solid dispersion of SiO_2_ in NR matrix was successfully improved by the addition of alginate as dispersion agent, resulting in no phase separation at high filler loading in NR matrix. The results agree with those of previous works on vulcanized rubber composites [32,33]; it was reported that the mechanical properties of rubber vulcanizates filled with WRHA were better than composites filled with BRHA.

Further crosslinking of the composites by CaCl_2_ significantly improved the tensile strength of NR–WRHA, especially at high WRHA loading content. After the immersion of NR composites in CaCl_2_ solution, calcium ion created gelation and ionic crosslinking with specific and strong interactions between Ca^2+^ with G blocks of alginate, forming the net structure [34]. This ionic crosslinking can improve the structural stability of the composite films and reinforce filler–filler and filler–rubber interactions, resulting in enhancement of the tensile strength of the composite film. After crosslinking sodium alginate/natural rubber/coconut composite by CaCl_2_, the composites were highly stable [35]. However, it was found that the mechanical properties of NR–BC films were reduced by crosslinking the composites by CaCl_2_. The different effects of CaCl_2_ crosslinking on NR–BRHA and NR–WRHA might be attributable to differences in the porous structure of those fillers. WRHA presents bimodal porous structure with higher porosity and higher pore sizes, resulting in better dispersion of CaCl_2_ into the mixtures and more interactions of fillers–alginate–NR of NR–W films. Consequently, alginate crosslinking with Ca^2+^ could enhance the tensile strength of NR–WC. A similar observation was also noted by Costa et al. [36]. The efficiency and rate constant of vulcanization of NR–BRHA was found to be lower than that of NR–WRHA vulcanization, because the pores of BRHA were too small for polymer chains to enter. Moreover, large internal surface areas might be a disadvantage, because a certain proportion of accelerator might become immobilized and inactivated. The adsorbed Ca^2+^ on surface might result in weakened filler–rubber interactions. Thus, mechanical properties of the NR–BC composite films were reduced by CaCl_2_ crosslinking.

The previous studies demonstrated the limitations of polar filler loading in NR composites. They found the tensile strength of the NR composite increased with increasing the filler content up to a maximum value (20–60 phr) and then decreased [12,13,31,32,37]. Higher filler loading in NR composite could lead to formation of filler agglomeration and undispersed filler in NR matrix, resulting in a weak filler–rubber interaction. RHA has high surface polarity and strong filler-filler interaction, which leads to filler agglomerates and poor RHA dispersion in NR matrix, resulting in low mechanical properties of NR composite. The present study demonstrates that the tensile strength of uncured NR composites increases with increasing BRHA and WRHA loading, and that the maximum tensile strength was obtained at BRHA/WRHA loading of 100 phr. The results indicate that the latex aqueous microdispersion process with dispersing agent is suitable for composite preparation to achieve homogeneous dispersion of RHA fillers in NR matrix. Sodium alginate is suitable for the use as a dispersing agent in this system, because it can improve RHA dispersion and reduce filler-filler interactions. Previously, sodium alginate as a dispersing agent was proven to improve the stability, filler dispersion and viscosity of composites of NR–coal fly ash [16], NR–sago starch [38] and NR–microfibrillated cellulose [39].

### 3.6. Water Absorption Capacity

**Figure 8:**
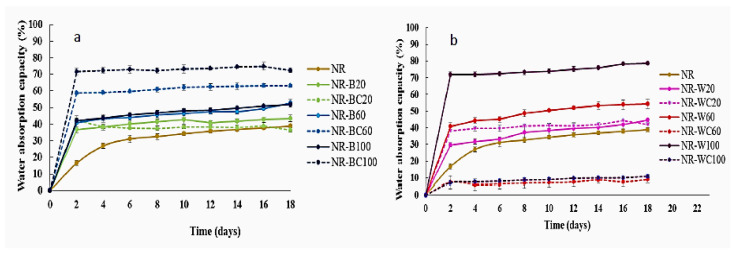
Water adsorption capacity of NR, NR–B, NR–BC (**a**) and NR–W, NR–WC (**b**).

In the water sorption and toluene sorption tests, the length and width of the film specimen were 2 cm and 2 cm. The thickness was 0.5–0.6 mm. The film thickness slightly increased with increasing filler content but was not significantly changed by CaCl_2_ crosslinking. The results of the water absorption capacity (WAC) tests of NR and NR composites filled with BRHA/WRHA are presented in Figure 8. The WAC of NR film was lower than NR–B and NR–W films, due to the hydrophobic nature of NR. The water adsorption of NR film was nonlinearly increased during a period of 0–6 days for water uptake of 0–31% and then was slightly increased during the immersion in water for 6–18 days. The water absorption behavior of filled polymer composites depended on the properties and nature of fillers such as functionality, polarity, specific surface area, filler loading and time in water [40]. The results demonstrate that the water absorption of NR–B, NR–BC and NR–W increase with increasing filler loading content, due to the hydrophilic nature of silica and other metal oxides, which are the main components in both ashes. The water uptake rates of the composite NR films filled with BRHA/WRHA were higher, as compared to NR film, and the saturation point was reached after the immersion in water for 2 days. The fillers, both BRHA and WRHA, are polar compounds and contain hydroxyl groups from silica and other metal oxides. The surface hydroxyl groups on metal oxides are sites for the adsorption of water molecules by hydrogen bonding. Thus, the increased RHA loading content promoted WAC of the composite films. The superior water absorption of NR composite filled with RHA was previously reported [40,41]. Without the CaCl_2_ crosslinking process, the composite film filled with WRHA demonstrated higher WAC than that filled with BRHA, which is likely due to the higher amount of SiO_2_ in WRHA, as compared to BRHA. The maximum WAC at around 78% was obtained from NR–W films at WRHA loading content of 100 phr.

**Figure 9:**
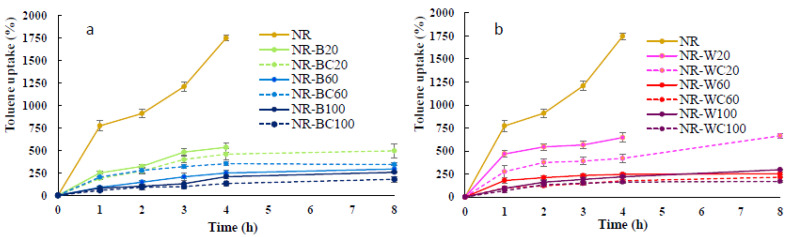
Toluene uptake of NR, NR–B, NR–BC (**a**) and NR–W, NR–WC (**b**).

The treatment by CaCl_2_ had different effects on the WAC of the NR composites filled with BRHA and WRHA. The CaCl_2_ crosslinked NR–WC showed considerably enhanced resistance for water uptake, especially at high WRHA loading content of 60 and 100 phr (Figure 9b). The water uptake of NR–WC at WRHA loading 100 phr was reduced to 11%, which was only ≈1/7 that of NR–W100 and was only ≈1/4 that of NR film. Previously, a significant reduction in water solubility of crosslinked alginate-based films by 1% CaCl_2_ was reported [34]. It was suggested that the crosslinking alginate by CaCl_2_ generated an “egg box” network. The COO– in the alginate binds to the Ca^2+^, resulting in reduced available alginate chains to bind with H_2_O molecules. The crosslinking NR–W composites by CaCl_2_ could also increase filler–rubber interactions and limited water diffusion into the NR–W composites. Accordingly, NR–WC presented lower WAC after crosslinking by CaCl_2_. On the other hand, the WAC of NR composite films filled with BRHA was increased after crosslinking by CaCl_2_ (Figure 8a). As previously discussed in the noneffective alginate crosslinking by Ca^2+^ of NR–BC composite films, in this case, the addition of CaCl_2_ was found to reduce filler–rubber interaction in NR–BC composites. The available COO– in alginate molecule and OH– in silica oxides could interact with water molecules, leading to higher water absorption of NR–BC. The addition of CaCl_2_ might also increase hydrophilicity of the films and, therefore, increase the water diffusion into NR–BC films.

### 3.7. Toluene Uptake

The results of the toluene uptake of NR and NR composite films soaked in toluene for 8 h are shown in Figure 9. The maximum toluene uptake of NR film was 1746% at the immersion in toluene for 4 h; after that, the film was decomposed in toluene. Toluene and NR are nonpolar; thus, this solvent can be highly absorbed in NR film. The high uptake rate and poor chemical resistance to nonpolar solvents of uncured NR were previously reported [16,17,42]. Uncrosslinked NR chains can dissolve in toluene, and the decrease in the solvent uptake of NR composites may reveal good filler—rubber interaction [42]. The toluene uptake rate of NR–B and NR–W composites at RHA loading of 20 phr was much less than that of NR film; however, after the immersion in toluene for 4 h, both NR–B and NR–W were, to some extent, decomposed in toluene. Uncured NR composites might be dissolved in nonpolar solvents, such as toluene [43]. More resistance to toluene uptake was observed in the NR composites with higher BRHA/WRHA content. The toluene uptake of the composite films decreased with increased filler loading and was considerably lower, as compared to that of pure NR film [16,39]. The low toluene uptake of NR–B and NR–W films was exhibited at RHA loading of 60–100 phr, which represented about 260–300% (or 0.15–0.17 of that of the NR film), due to polar nature of silica oxides, the major constituent in both RHAs. The integration of polar fillers into NR matrix can reduce the toluene uptake rate and adsorption capacity. In addition, the NR composites filled with BRHA/WRHA at 60–100 phr maintained their structural stability during immersion in toluene for 8 h. This observation indicates that the composite films had good dispersion of RHA in the NR matrix.

After crosslinking by CaCl_2_, the NR composites filled with WRHA/BRHA demonstrated slightly improved resistance to toluene. The toluene uptakes of NR–BC and NR–WC were reduced to 170–180%. The crosslinked films had better stability in toluene, as no decomposition of the films was observed during immersion in toluene for 8 h. Thus, the CaCl_2_ treatment seems to improve the solvent resistance of composite films. The addition of calcium ions into NR matrix possibly makes the surface more hydrophilic, resulting in lower diffusion of nonpolar solvent into NR matrix. The lowering of toluene uptake might also be attributable to the good interaction between filler and NR matrix [44].

### 3.8. Biodegradation in Soil

**Figure 10:**
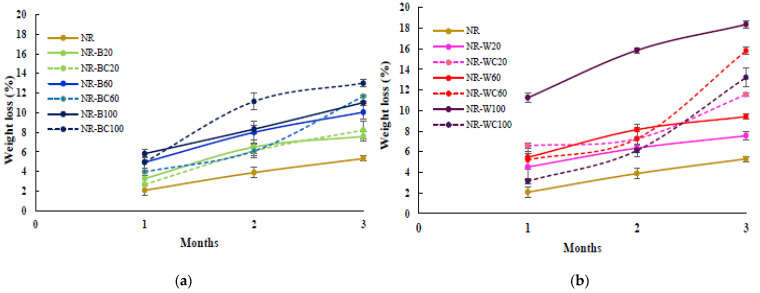
Preliminary test for biodegradation in soil of NR, NR–B, NR–BC (**a**) and NR–W, NR–WC (**b**).

**Figure 11:**
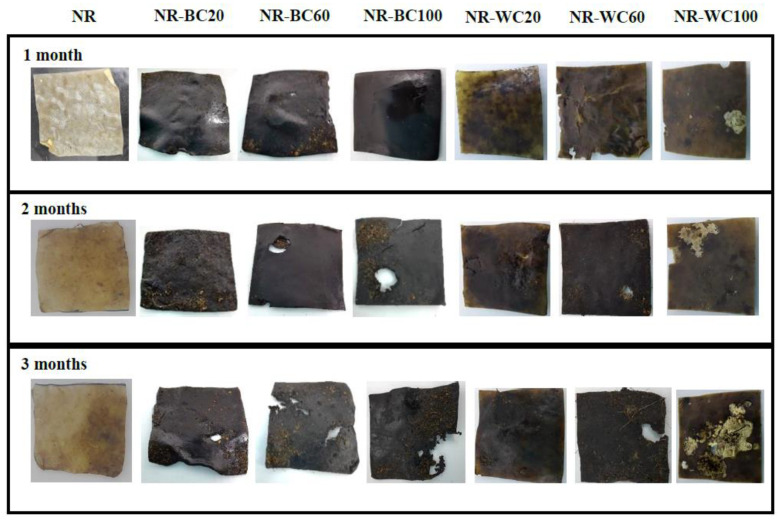
The visual analysis of biodegradation in soil of NR–BC and NR–WC for 3 months.

The preliminary study of biodegradation in soil of pure NR film and NR composite films filled with RHAs was determined in uncontrolled conditions (with an average temperature of ≈30 °C). The percentage weight loss and the visual analysis of the biodegraded samples after 1, 2 and 3 months are shown in Figure 10 and Figure 11, respectively. The weight loss of the pure NR film was lower than all NR composite films at any degradation time, due to the slow degradation of NR in natural environments by microorganisms [45]. After 3 months, the weight loss of NR, NR–BRHA and NR–WRHA were 5.3%, 7.6–13.0% and 7.6–18.4%, respectively. Overall, the NR–BRHA composite presented comparable weight loss to NR–WRHA composites at similar RHA loading contents, except for the NR composites with 100 phr RHA loadings, of which, NR–W100 demonstrated relatively higher biodegradability in soil. The weight loss of composites increased with increased filler loading in all conditions. These results agree with those of Ramasamy et al. [46], who reported that the weight loss percentage of NRL foam increased with increased rice husk powder, because, at lower rice husk powder loading in foam composite, microbial activity was not high due to lack of microbial growth supports.

The effect of the CaCl_2_ treatment of the NR composites was observed from the composites at filler loading of 100 phr in both cases but in opposite directions. The weight loss of NR–BC100 was higher than that of NR–B100, whereas the weight loss of NR–WC100 was lower than NR–W100. These results are in accord with those on the water absorption capacity, which can be explained by the high number of fillers in NR matrix generating hydrophilic surfaces, resulting in enhanced water absorption of the composites. The higher water content in NR matrix could promote microbial activities that increase the rate of degradation [31,47]. The weight loss of NR–WC100 was lower than NR–W100, because the CaCl_2_ crosslinked film displayed a higher tensile strength with lower WAC, as compared to NR–W100. On the other hand, the weight loss of NR–BC 100 was higher than NR–B100, due to the higher WAC of the composite after CaCl_2_ crosslinking, as discussed earlier. It was also suggested that the use of sodium alginate as a dispersing agent in NR composites could increase the rate of biodegradation of the composites [38]. Figure 11 presents visual analysis of the biodegradation of NR and NR composite films during a period of 1–3 months. The enhanced degradation of NR composite films filled with higher RHA loading was clearly observed, especially at 3 months of biodegradation in soil. Black spots were found in the composite films reinforced with WRHA, while white spots, as well as fogging on film surfaces, were observed after 1 month in soil, indicating soil microflora activities in the composites, which is a good indicator of biodegradation [48]. Moreover, after 3 months in soil, some parts of the composite films disappeared. The results revealed that NR–BRHA and NR–WRHA could be biodegradable in soil.

### 3.9. Dielectric Properties

**Figure 12:**
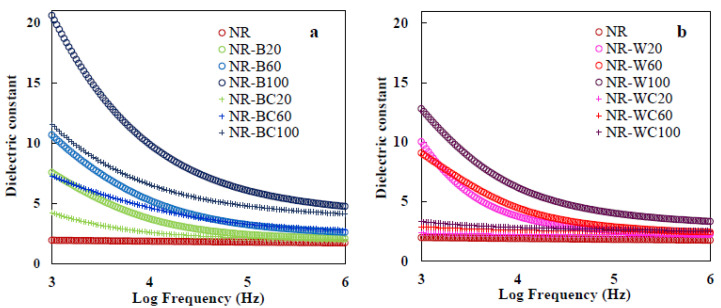

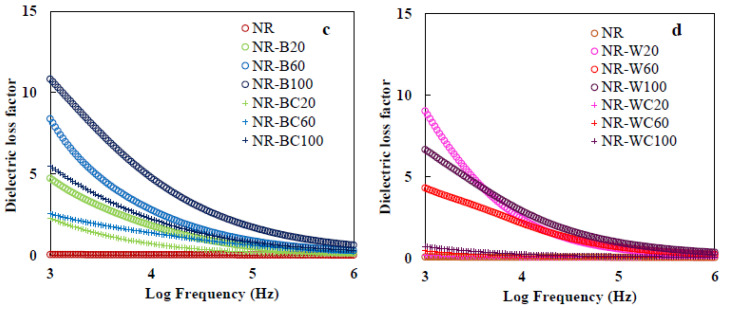
Dielectric constant of (**a**) NR, NR–B, NR–BC and (**b**) NR, NR–W, NR–WC and dielectric loss of (**c**) NR, NR–B, NR–BC and (**d**) NR, NR–W, NR–WC.

Dielectric properties of materials are important characteristics for predicting behavior with respect to application in electronic materials, such as print circuit boards, power transformers, microelectronics, radio propagation, remote sensing and actuator applications [15]. The dielectric properties of polymers depend on cations, dipole moment and space charge polarizations [49]. On the other hand, dielectric behavior of composite materials depends on dielectric properties of the matrix and filler, chemical composition, structure, shape and morphology of particles and filler dispersion in the composite [50]. The dielectric constant is the amount of energy from an external electrical field stored in materials. The dielectric constant of NR–BRHA and NR–WRHA composites at different filler loadings are presented in Figure 12a,b. The frequency dependence of the dielectric constant at ambient temperature was analyzed. The NR film had a low dielectric constant, in the range of 1.8–2, attributable to the nonpolar nature of NR, which has only instantaneous electronic and atomic polarization [51]. It was demonstrated that the dielectric constant of the NR composite films filled with RHAs increased with increased BRHA/WRHA loading at all frequencies. BRHA and WRHA contained 87 and 95% silica oxides with polar hydroxyl groups of silanol (Si–OH) on surface. Silica as a dielectric metal oxide semiconductor has a dielectric constant of about 3–9, and it has been used in micro- and nano-electronic industries [52]. Thus, the increase of RHA in NR composites resulted in higher dipole or orientation polarization; thus, the dielectric constant was enhanced [51]. In addition, the increase in RHA loading resulted in an increased space charge, leading to polarization in the composite matrix [53]. It was shown that the NR composite films presented a higher dielectric constant at lower frequencies. At high frequency, molecular movements arrested or decreased orientation polarization that leads to the decreased value of the dielectric constant [53,54]. The dielectric constants of NR–BRHA were relatively higher than those of NR–WRHA, which should be due to the higher degree of crystallinity of BHRA, as compared to that of WRHA. Correlations between crystal structure and dielectric properties of metal oxides were reported [55]. Metal and carbon-based filler could be acting as charge centers and increasing segmental mobility in the polymer matrix, which would enhance the dielectric constant of composites [56,57]. Oxides are suggested as promising materials in high dielectric materials, as they possess both high dielectric constants and large band gaps [55].

The crosslinking of the composite films by CaCl_2_ caused a considerable drop in dielectric properties in all cases. The addition of CaCl_2_ might reduce the amount of electron charges, including COO– (from alginate) and OH– (from silica), in the composite films. The higher number of free protons suggests more localization of charge carriers along with mobile ions, resulting in higher dipole polarization and a higher dielectric constant [54]. For this reason, the value of the dielectric constant of composite film after crosslinking alginate was much lower than that of films without CaCl_2_ treatment. The obtained values for the dielectric constants of the films from the lowest value to the highest value were NR < NR–WC < NR–BC < NR–W < NR–B. These results could also imply that the stronger interactions between WRHA filler and NR matrix could lower the hydrophilicity and polarity of the composite films. A similar effect from the reinforcement that caused the reduction of orientation polarization and the dielectric constant was previously reported [51].

The dielectric loss factor presents the amount of energy loss from a material due to an external electric field [58]. The frequency dependence of dielectric loss of composites is shown in Figure 12c,d. The values of dielectric loss demonstrate similar trends as those of the dielectric constant, which increased with increased filler loading and declined with increases in frequency. The increase of RHA loading enhanced the polar groups in the composite films, which led to enlargement of orientation polarization and relaxation in composite, resulting in relatively high energy loss [53]. In addition, increasing amorphous phase to the composite enhanced the flow of current through the amorphous region and generated amorphous phase relaxation, resulting in higher dielectric loss [51,59]. At low frequency, dielectric loss was high, because of interfacial polarization enhanced by the difference between the conductivity of various phases [53]. This result agrees with the previous work by Jamal et al. [57], in which the dielectric loss of nickel–rubber nanocomposites was high at frequency lower than 105 Hz, due to relaxation time of pure rubber enhanced when nickel nanoparticles were incorporated into the matrix. Moreover, the results indicate dielectric loss factor of NR–WC composites was lower than that of other composites at all WRHA loadings (~0.7–2.1 at frequency 103 Hz). The effect of crosslinking composite by CaCl_2_ on the reduction of the dielectric loss factor was the same as that on the dielectric constant for similar reasons. Less free space in the matrix resulted in a low dielectric loss factor [54].

## 4. Conclusions

BRHA/WRHA-filled uncured NR composites were successfully prepared via a latex aqueous microdispersion process with the use of sodium alginate as a dispersing agent. SEM images presented good dispersion of RHAs in composite matrices and no phase separation with RHA loading in the range of 20–100 phr. The filler–rubber interactions of composites were observed via FTIR analysis. The effective crosslinking of alginate in the composites by CaCl_2_ was achieved on NR–WC films. The mechanical properties of NR composite films were improved by BRHA/WRHA reinforcement. NR–WC at filler loading of 100 phr presented the highest tensile strength and the lowest water and toluene uptake. The results indicate that crosslinking NR–W composite by CaCl_2_ promotes filler–alginate–NR interaction, mechanical properties and structural stability of NR–WC films. In addition, the preliminary biodegradation in soil for 3 months revealed relatively improved biodegradability with RHA loading. The weight losses of NR–Bs and NR–Ws after 3 months in soil were about 7.6–13.0% and 7.6–18.4%, respectively. Moreover, the reinforcement considerably improved dielectric properties of the composite films. Since the NR composites filled with RHAs exhibited improved properties in terms of mechanical properties, chemical resistance, biodegradability and dielectric properties, they have potential for further development as stretchable conductive substrate or semiconducting polymer films for electronic applications.

## Figures and Tables

**Table 1:** Chemical compositions of black rice husk ash (BRHA) and white rice husk ash (WRHA).

Chemical Compositions	BRHA (%wt)	WRHA(%wt)
SiO_2_	87.00	95.30
Al_2_O_3_	2.51	1.76
K_2_O	1.07	0.89
CaO	0.52	0.71
P_2_O_5_	0.63	0.57
MgO	0.32	0.36
Fe_2_O_3_	0.16	0.13
SO_3_	-	0.17
MnO	719 ppm	743 ppm
ZnO	64.1 ppm	113 ppm
Rb_2_O	44.4 ppm	-
Loss on ignition	7.783	0.105

**Table 2:** The surface area, pore volume and average pore diameter of BRHA and WRHA.

Rice Husk Ash	BET Surface Area (m^2^/g)	Pore Volume (cm^3^/g)	Average Pore Diameter (nm)
BRHA	51.57	0.24	9.22
WRHA	40.06	0.48	23.92

## Data Availability

The data presented in this study are available on request from the corresponding author.
